# Commensal Protection of *Staphylococcus aureus* against Antimicrobials by *Candida albicans* Biofilm Matrix

**DOI:** 10.1128/mBio.01365-16

**Published:** 2016-10-11

**Authors:** Eric F. Kong, Christina Tsui, Sona Kucharíková, David Andes, Patrick Van Dijck, Mary Ann Jabra-Rizk

**Affiliations:** a Graduate Program in Life Sciences, Molecular Microbiology and Immunology Program, University of Maryland, Baltimore, Maryland, USA; b Department of Microbiology and Immunology, School of Medicine, University of Maryland, Baltimore, Maryland, USA; c Department of Oncology and Diagnostic Sciences, Dental School, University of Maryland, Baltimore, Maryland, USA; d Laboratory of Molecular Cell Biology, KU Leuven, Leuven-Heverlee, Flanders, Belgium; e Department of Molecular Microbiology, VIB, Leuven-Heverlee, Flanders, Belgium; f Department of Medicine, Microbiology and Immunology, University of Wisconsin, Madison, Wisconsin, USA

## Abstract

Biofilm-associated polymicrobial infections, particularly those involving fungi and bacteria, are responsible for significant morbidity and mortality and tend to be challenging to treat. *Candida albicans* and *Staphylococcus aureus* specifically are considered leading opportunistic fungal and bacterial pathogens, respectively, mainly due to their ability to form biofilms on catheters and indwelling medical devices. However, the impact of mixed-species biofilm growth on therapy remains largely understudied. In this study, we investigated the influence of *C. albicans* secreted cell wall polysaccharides on the response of *S. aureus* to antibacterial agents in biofilm. Results demonstrated significantly enhanced tolerance for *S. aureus* to drugs in the presence of *C. albicans* or its secreted cell wall polysaccharide material. Fluorescence confocal time-lapse microscopy revealed impairment of drug diffusion through the mixed biofilm matrix. Using *C. albicans* mutant strains with modulated cell wall polysaccharide expression, exogenous supplementation, and enzymatic degradation, the *C. albicans*-secreted β-1,3-glucan cell wall component was identified as the key matrix constituent providing the bacteria with enhanced drug tolerance. Further, antibody labeling demonstrated rapid coating of the bacteria by the *C. albicans* matrix material. Importantly, via its effect on the fungal biofilm matrix, the antifungal caspofungin sensitized the bacteria to the drugs. Understanding such symbiotic interactions with clinical relevance between microbial species in biofilms will greatly aid in overcoming the limitations of current therapies and in defining potential new targets for treating polymicrobial infections.

## IMPORTANCE

The fungus *Candida albicans* and the bacterium *Staphylococcus aureus* are important microbial pathogens responsible for the majority of infections in hospitalized patients and are often coisolated from a host. In this study, we demonstrated that when grown together, the fungus provides the bacterium with enhanced tolerance to antimicrobial drugs. This process was mediated by polysaccharides secreted by the fungal cell into the environment. The biofilm matrix formed by these polysaccharides prevented penetration by the drugs and provided the bacteria with protection. Importantly, we show that by inhibiting the production of the fungal polysaccharides, a specific antifungal agent indirectly sensitized the bacteria to antimicrobials. Understanding the therapeutic implications of the interactions between these two diverse microbial species will aid in overcoming the limitations of current therapies and in defining new targets for treating complex polymicrobial infections.

## INTRODUCTION

Polymicrobial infections caused by a combination of microorganisms are responsible for significant mortality and morbidity, particularly those associated with biofilms formed on indwelling medical devices (1–3). Biofilms are structured three-dimensional communities of surface-associated microbial populations embedded in a matrix of extracellular polysaccharides, proposed to provide a structural scaffold and protection for biofilm cells (4–6). Therefore, in a biofilm, microbes are afforded a stable environment and can tolerate high concentrations of antimicrobials. The impact of these biofilms on public health is dramatic, as cells released from biofilms can migrate into the bloodstream and cause systemic infections with high mortality (7). Importantly, the increase in drug resistance has provided a strong impetus to understand the mechanisms of the enhanced tolerance of biofilm-associated infections to antimicrobial therapy and particularly polymicrobial infections. Although mixed fungal-bacterial infections tend to be the most complex and challenging to treat, the impact of these interactions on therapy remains largely understudied.

Among the fungal species, *Candida albicans* is the most common human pathogen, causing diseases ranging from superficial mucosal to life-threatening systemic infections (8–10). The ability of *C. albicans* to transition from commensal to pathogen is primarily the result of its aptitude for morphologically switching between yeast and hyphal forms (9, 11). In fact, the majority of *C. albicans* infections are associated with its ability to form biofilms, where adhesion of yeast cells to the substrate is followed by proliferation and hypha formation, resulting in a network of cells embedded in a matrix (7, 12, 13). *Candida albicans* biofilm matrix is complex, with major polysaccharide constituents being α-mannan, β-1,6-glucan, and β-1,3-glucan (14, 15). Although a relatively minor component, β-1,3-glucan is considered the critical matrix polysaccharide, as extracellular glucan has been linked to biofilm resistance to antifungals (16, 17). In fact, previous studies have shown elevated β-1,3-glucan levels to be characteristic of biofilm cells both in the fungal cell walls and as a secreted form. Of more significance, the increase in β-1,3-glucan secretion by biofilm cells was shown *in vivo* in animal models of catheter infection and disseminated candidiasis (12). Glucan synthase Fks1p is responsible for the synthesis of cell wall β-1,3-glucan during biofilm growth, and *FKS1* disruption was shown to reduce manufacture and deposition of β-1,3-glucan in the biofilm matrix (18). Importantly, using strains with modulated *FKS1* expression, a study by Nett et al. (18) demonstrated that reduction in expression rendered biofilms more susceptible to various antifungals, whereas overexpression resulted in increased resistance.

In various niches in the host, *C. albicans* coexists with various bacterial species, including *Staphylococcus aureus* (2, 19–21). Although *S. aureus* primarily exists as a commensal organism, this bacterial pathogen is implicated in a variety of diseases ranging from minor skin infections to more serious invasive diseases and specifically device-associated biofilm infections (22–24). With the emergence of methicillin-resistant *S. aureus* (MRSA), this ubiquitous pathogen is becoming an even greater therapeutic challenge (25, 26). *S. aureus* is a poor former of biofilms; however, together with *C. albicans*, this species forms a substantial biofilm where the fungus creates a scaffold for the bacteria (27–29). Our previous *in vitro* studies have demonstrated that *S. aureus* exhibits high affinity to the *C. albicans* hyphal form, as these species coadhere and interact synergistically in a biofilm. Further, our studies identified the *C. albicans* hypha-specific adhesin Als3p to be involved in the coadherence process (27).

The growing use of implanted medical devices is another reason why the incidence of *Candida* and staphylococcal infections has steadily increased, since the majority of these infections are emerging from biofilms formed on medical implants (10, 13, 23, 30, 31). In fact, a recent analysis of cases of endocarditis associated with an implanted device found ~25% of the infections to be polymicrobial, and in another study, 27% of nosocomial *C. albicans* bloodstream infections were estimated to be polymicrobial (32, 33). Importantly, *S. aureus* was found to be the third most commonly coisolated species with *C. albicans* (33). Although numerous studies have reported the coisolation of *C. albicans* and *S. aureus* from a multitude of diseases such as periodontitis, denture stomatitis, cystic fibrosis, keratitis, ventilator-associated pneumonia, and urinary tract catheter and burn wound infections, the clinical significance of their interaction in a host remains largely understudied, likely due to lack of suitable animal models (34–38). However, using a mouse model of oral infection, we recently demonstrated that upon onset of oral candidiasis (thrush), mice cocolonized with *C. albicans* and *S. aureus* suffered systemic bacterial infection with high morbidity and mortality (39).

The inherent characteristics of biofilms are multifactorial but are largely due to the extracellular matrix encasing the biofilm cells, which prevents drugs and other stresses from penetrating the biofilm. Therefore, *S. aureus* and *C. albicans* interactions in mixed biofilm infections may also impact response to antimicrobial therapy (39). Although enhanced *in vitro* tolerance to antimicrobials in *C. albicans* and staphylococcus mixed biofilms has been reported, the mechanism and specific factors behind these observations remain undefined (40, 41). To that end, in this study, experiments were designed to demonstrate the impact of *C. albicans* biofilm matrix and secreted components on the susceptibility of *S. aureus* to antibacterial drugs in biofilm, focusing on vancomycin, the drug of choice for treatment of MRSA infections. The overall goal of this study is to provide crucial insights into the enhanced tolerance of biofilm-associated polymicrobial infections to antimicrobial therapy.

## RESULTS

### Comparative assessment of the susceptibility of *S. aureus* to vancomycin in single and mixed biofilms with *C. albicans*.

**FIG 1:**
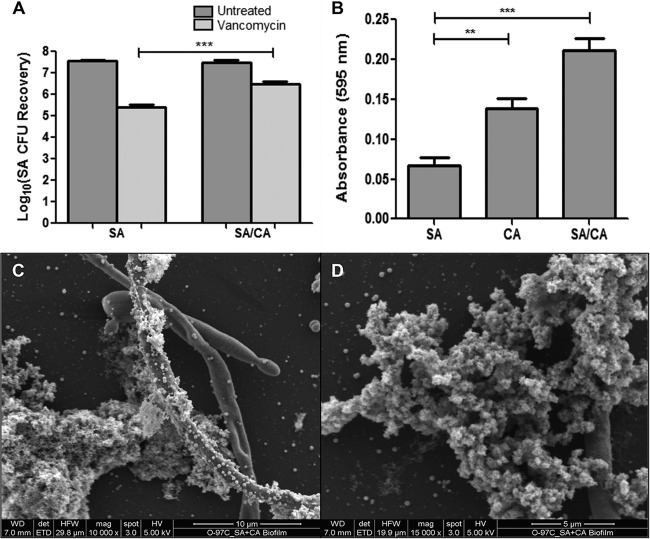
Assessment of relative biomass and vancomycin susceptibility in single *S. aureus* (SA) and mixed *S. aureus* and *C. albicans* (SA/CA) biofilms. (A) Preformed (24-h) *S. aureus* single and mixed biofilms were treated with vancomycin (800 µg/ml) for an additional 24 h. CFU recovery of *S. aureus* from both biofilms showed a significant increase in *S. aureus* recovery from mixed biofilms following vancomycin treatment (***, *P* < 0.001). (B) To assess biomass, 24-h single- and mixed-species biofilms were treated with crystal violet. Based on absorbance (595 nm), results demonstrated significantly higher biomass for mixed biofilms than for *S. aureus* biofilm (**, *P* < 0.01; ***, *P* < 0.001). Means and standard errors of the means are shown. (C and D) These results were corroborated by SEM analysis where images demonstrated adherence to and clumping of *S. aureus* around *C. albicans* hyphae, forming thick biofilm aggregates.

The impact of mixed-species biofilm growth on *S. aureus* susceptibility to vancomycin was assessed following 24-h treatment of preformed single and mixed biofilms. Based on CFU, results demonstrated a significant increase in *S. aureus* survival in mixed biofilms following vancomycin treatment compared to survival in single biofilm (Fig. 1A). Analysis of biofilms using crystal violet staining indicated a significant increase in biofilm biomass in mixed biofilms relative to *S. aureus* biofilm (Fig. 1B). These results were corroborated by scanning electron microscopy (SEM) analysis where images of mixed biofilms revealed a thick matrix, with *S. aureus* adhering to and forming aggregates around the *C. albicans* hyphae (Fig. 1C and D).

### Exogenous supplementation of *S. aureus* biofilms with *C. albicans* biofilm matrix material enhances *S. aureus* tolerance to vancomycin.

**FIG 2:**
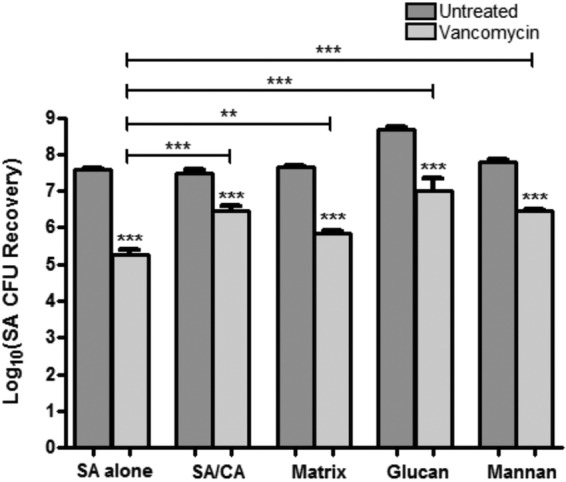
Effect of *C. albicans* matrix polysaccharides on *S. aureus* (SA) susceptibility to vancomycin. *S. aureus* single biofilms were allowed to form in the presence of purified *C. albicans* (CA) biofilm matrix material (0.02 mg/liter), glucan (1 mg/ml), or mannan (1 mg/ml). *S. aureus* dual-species biofilms were grown with *C. albicans* (SA/CA). Preformed biofilms were treated with vancomycin (800 µg/ml) for 24 h. *S. aureus* CFU recovery from biofilms demonstrated significant increase in *S. aureus* tolerance to vancomycin in the presence of matrix material, glucan, and mannan. Susceptibility to vancomycin was assessed by CFU counts and corroborated by an MTS assay (**, *P* < 0.01; ***, *P* < 0.001). Means and standard errors of the means are shown.

To isolate the role of *C. albicans* biofilm matrix in the enhanced tolerance of *S. aureus* to vancomycin in mixed biofilms, purified matrix material recovered from *C. albicans* biofilms was incorporated in susceptibility testing. Assessment of *S. aureus* viability by the MTS [3-(4,5-dimethylthiazol-2-yl)-5-(3-carboxymethoxyphenyl)-2-(4-sulfophenyl)-2H-tetrazolium] metabolic assay and CFU recovery demonstrated a significant increase in *S. aureus* survival in single biofilms formed in the presence of matrix material (Fig. 2). In order to identify the contribution of specific matrix components, similar experiments were also performed where *S. aureus* biofilms were allowed to form in the presence of glucan or mannan. Results from these experiments demonstrated a comparable increase in survival with vancomycin.

### Differential *C. albicans* β-1,3-glucan but not α-mannan expression modulates vancomycin tolerance in mixed biofilms.

**FIG 3:**
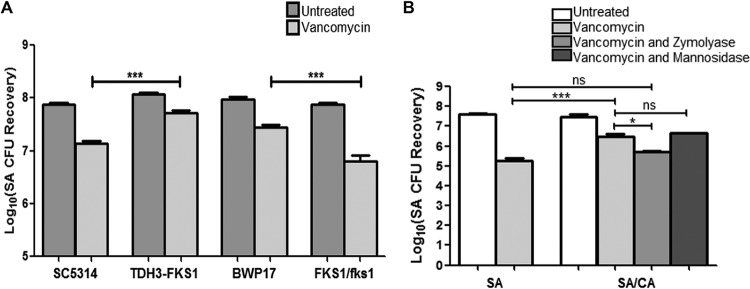
Effect of *C. albicans* FKS1 modulation and Zymolyase treatment on *S. aureus* (SA) susceptibility to vancomycin. (A) Preformed *S. aureus* single- and mixed-species biofilms with the various *C. albicans* strains were treated for 48 h with vancomycin (800 µg/ml). Based on CFU recovery, a significant increase in *S. aureus* survival was seen when grown in mixed biofilms with the *TDH3-FKS1* glucan-overexpressing strain compared to its parent strain SC5314, whereas a significant decrease in survival was noted when *S. aureus* was grown with the glucan-deficient heterozygous *FKS1/fks1* mutant strain compared to its parental strain BWP17 (***, *P* < 0.0001). (B) *S. aureus* single (SA) and mixed (CA/SA) biofilms were allowed to form for 24 h in the presence of Zymolyase (5 U/ml) or α-mannosidase (2 U/ml) and then treated with vancomycin (800 µg/ml) for 24 h. Based on CFU recovery, although growth with *C. albicans* in mixed biofilms provided *S. aureus* with a significant increase in survival with vancomycin, the increase in tolerance was diminished upon treatment with Zymolyase but not with α-mannosidase (*, *P* < 0.05; ***, *P* < 0.0001; ns, not significant). Means and standard errors of the means are shown.

The key role for the β-1,3-glucan matrix component was demonstrated using *C. albicans* strains with modulated β-1,3-glucan expression in mixed biofilms treated with vancomycin. Compared to their respective reference strains, *S. aureus* recovery following vancomycin treatment was consistently higher in the presence of the *TDH3-FKS1* glucan-overexpressing strain but lower when grown with the *FKS1/fks1*Δ glucan-deficient heterozygous mutant strain (Fig. 3A). No impact on *S. aureus* vancomycin susceptibility was seen in mixed biofilms with the mannosylation mutants or the *zap1*Δ*/zap1*Δ mutant lacking the zinc response transcription factor Zap1 (a negative regulator of β-1,3-glucan) (data not shown).

### β-1,3-Glucanase but not α-mannosidase treatment of mixed biofilms abrogates the enhanced tolerance to vancomycin.

To demonstrate the importance of the *C. albicans* matrix polysaccharides, experiments were also performed where single- and mixed-species biofilms were treated with vancomycin in the presence of the glucan-degrading enzyme Zymolyase or the mannan-degrading enzyme α-mannosidase. Based on CFU recovery, the increase in *S. aureus* vancomycin tolerance in mixed biofilms was significantly diminished upon Zymolyase treatment, to a level comparable to that for *S. aureus* single-species biofilm treated with vancomycin. However, no significant differences were noted with the α-mannosidase treatment (Fig. 3B). Since Zymolyase is known to have contaminating protease activity, enzymatic digestion experiments were also performed using proteinase K; results from these experiments demonstrated no effect for proteinase K on *S. aureus* response to vancomycin (data not shown).

### SEM of *S. aureus* biofilm architecture supplemented with *C. albicans* biofilm matrix material and exogenous β-1,3-glucan.

**FIG 4:**
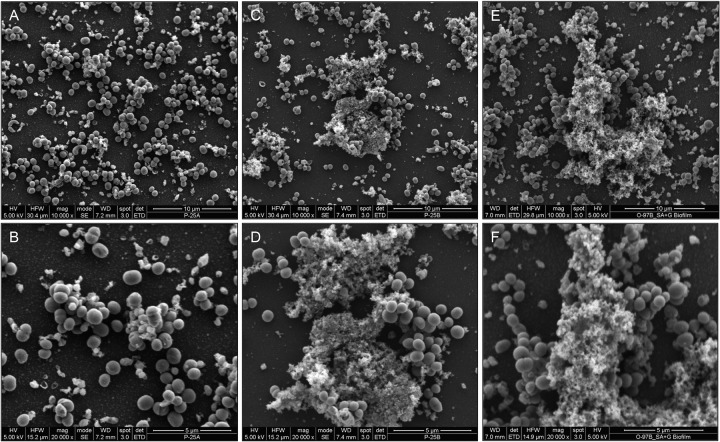
Effect of purified matrix and glucan supplementation on *S. aureus* biofilm architecture using SEM analysis. (A and B) Images of *S. aureus* biofilms demonstrated a thin and heterogeneous biofilm formed following a 48-h incubation. (C to F) In contrast, when grown in the presence of exogenous matrix material (0.5 mg/ml) (C and D) or glucan (0.25 mg/ml) (E and F), significant increases in biofilm matrix, mass, and bacterial cell clumping were seen.

To visualize the structure of the *S. aureus* biofilm supplemented with *C. albicans* matrix material, *S. aureus* biofilms were allowed to form for 24 h in the absence and presence of purified matrix material or glucan, and the formed biofilms were comparatively examined by SEM analysis. Images revealed that where nonsupplemented *S. aureus* biofilms appeared thin and heterogeneous (Fig. 4A and B), growth in the presence of matrix material (Fig. 4C and D) or β-1,3-glucan (Fig. 4E and F) resulted in significantly increased biofilm mass with considerable aggregation of bacterial cells.

### *C. albicans* spent culture medium provides *S. aureus* with enhanced tolerance to vancomycin in single-species biofilm.

**FIG 5:**
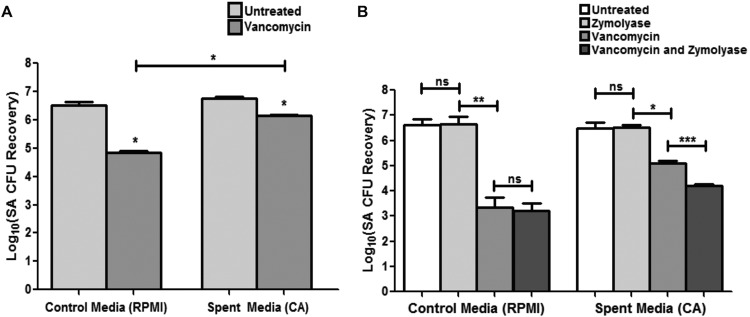
Effect of secreted effectors in *C. albicans* spent medium on *S. aureus* (SA) vancomycin susceptibility. (A) Supernatant from *C. albicans* culture medium (spent medium) was collected and used in *S. aureus* vancomycin susceptibility testing assays. Based on results from *S. aureus* CFU recovery, growth in *C. albicans* spent medium significantly enhanced *S. aureus* vancomycin tolerance compared to growth in control fresh medium (*, *P* < 0.05). (B) Since *C. albicans*-produced glucan is secreted into the medium, spent medium was treated with Zymolyase prior to vancomycin susceptibility testing. No significant effect for Zymolyase alone on *S. aureus* viability was seen; however, *S. aureus* tolerance to vancomycin was significantly diminished when spent medium was treated with Zymolyase. Results were corroborated with an MTS assay (*, *P* < 0.05; **, *P* < 0.001; ***, *P* < 0.0001; ns, not significant). Means and standard errors of the means are shown.

Since *C. albicans* cell wall polysaccharides are secreted, experiments were performed where *C. albicans* spent biofilm culture medium was recovered and used in vancomycin susceptibility testing of *S. aureus* single biofilms. Results from these experiments demonstrated that *C. albicans* cell-free spent medium provided *S. aureus* with significantly enhanced tolerance to vancomycin in the absence of *C. albicans* in the biofilm (Fig. 5A). To identify the secreted component conferring the tolerance, the spent medium was enzymatically pretreated with Zymolyase or α-mannosidase prior to use in vancomycin susceptibility assays. Results from these experiments demonstrated that, whereas Zymolyase treatment considerably diminished the observed protection in spent medium (Fig. 5B), α-mannosidase treatment had no significant impact on *S. aureus* susceptibility to vancomycin (data not shown). Zymolyase and α-mannosidase treatment alone did not have any effect on *S. aureus* viability.

### Inhibition of β-1,3-glucan synthesis in *C. albicans* compromises the enhanced tolerance to vancomycin in mixed biofilms and of spent medium.

**FIG 6:**
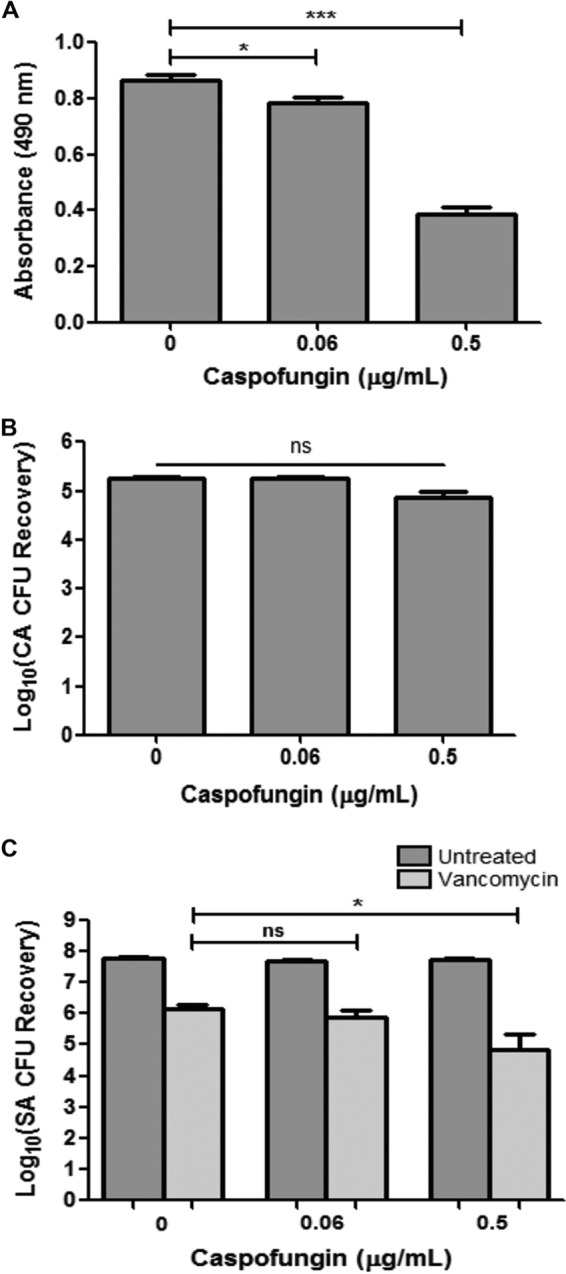
Effect of caspofungin treatment of mixed biofilms on response of *S. aureus* (SA) to vancomycin. (A) In order to determine the *C. albicans* (CA) sublethal, inhibitory concentration of caspofungin, susceptibility assays were performed where preformed *C. albicans* biofilms were treated with caspofungin for 24 h. Results from the MTS assay indicated a concentration-dependent decrease in *C. albicans* metabolic activity with caspofungin. (B) However, no significant effect on *C. albicans* viability was noted for these concentrations based on CFU enumeration (*, *P* < 0.05; ***, *P* < 0.0001; ns, not significant). (C) Mixed species biofilms were similarly treated with caspofungin and vancomycin (800 µg/ml), and *S. aureus* viability was assessed by CFU recovery. Results demonstrated a significant decrease in *S. aureus* survival with vancomycin when mixed biofilms were treated with 0.5 µg/ml caspofungin (*, *P* < 0.05). Means and standard errors of the means are shown.

**FIG 7:**
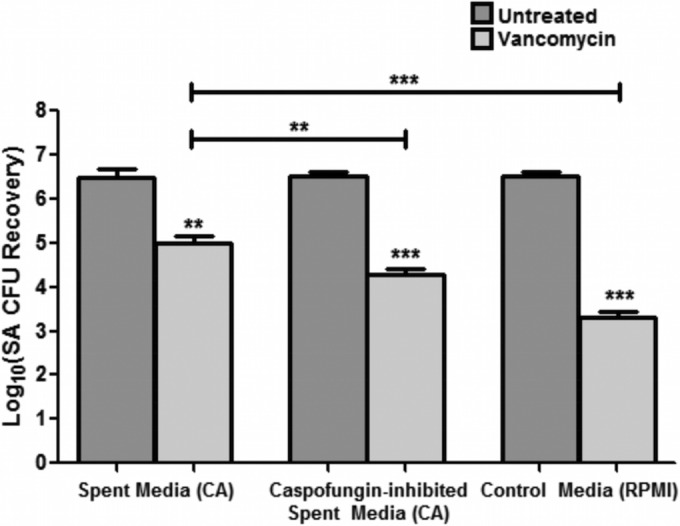
Effect of caspofungin treatment of *C. albicans* (CA) biofilms on ability of spent culture medium to confer vancomycin tolerance. *C. albicans* biofilms were treated with caspofungin (0.5 µg/ml) in order to diminish glucan synthesis and secretion. Recovered spent culture medium was then used in vancomycin (400 µg/ml) susceptibility testing of *S. aureus* (SA) single-species biofilms. Based on *S. aureus* CFU recovery, results demonstrated that caspofungin treatment of *C. albicans* biofilms significantly diminished the ability of the spent medium to provide *S. aureus* with protection against vancomycin. Results were also corroborated by an MTS assay (**, *P* < 0.01; ***, *P* < 0.001). Means and standard errors of the means are shown.

In addition to enzymatic degradation, the antifungal caspofungin, an inhibitor of β-1,3-glucan synthesis in the fungal cell, was used to assess whether its effect on cell wall glucan synthesis impacts *S. aureus* vancomycin susceptibility. Prior to experiments, the subinhibitory caspofungin concentration on *C. albicans* was determined using the MTS assay and CFU enumeration to determine its effect on metabolic activity and viability, respectively. Results from the MTS assay indicated a concentration-dependent decrease in *C. albicans* metabolic activity with caspofungin; however, no effect on viability was noted for these concentrations (Fig. 6A and B). The selected subinhibitory caspofungin concentration (0.5 µg/ml) was subsequently used in mixed biofilm vancomycin susceptibility assays. Based on CFU recovery, results demonstrated a significant decrease in *S. aureus* survival with vancomycin when mixed biofilms were treated with caspofungin (Fig. 6C). Further, experiments were also performed to determine whether the effect of caspofungin on β-1,3-glucan synthesis affects its secretion and, thus, the protective effect of *C. albicans* spent medium on *S. aureus*. Therefore, experiments were also performed where formed *C. albicans* biofilms were treated with caspofungin prior to recovering the spent culture medium, which was subsequently used in vancomycin susceptibility testing. Results from these experiments similarly demonstrated that caspofungin treatment of *C. albicans* biofilms significantly diminished the ability of recovered spent medium to confer protection against vancomycin on *S. aureus* (Fig. 7). Caspofungin did not have any effect on *S. aureus*.

### Impeded vancomycin diffusion through *S. aureus* biofilms grown with *C. albicans* or its purified and secreted matrix material.

**FIG 8:**
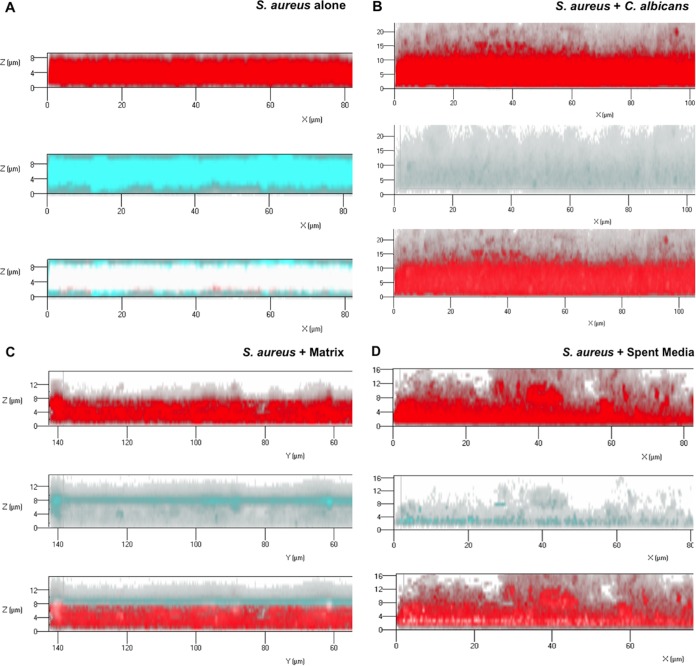
Representative CSLM images assessing diffusion of fluorescently labeled vancomycin through *S. aureus* grown with *C. albicans* or its derived biofilm matrix material or in spent medium. *S. aureus* biofilms were grown for 24 h with *C. albicans* or supplemented with 0.5 mg/ml of purified matrix material. *S. aureus* biofilms were also grown in *C. albicans* spent culture medium containing secreted matrix components. Biofilms were stained with ConA for biofilm matrix (red) and fluorescent vancomycin compound (cyan). Following a 1-h diffusion, the biofilms were visualized using CSLM. (A) In the *S. aureus* monospecies biofilms, vancomycin fully penetrated and diffused throughout the biofilm matrix (white; red/cyan merged), reaching the basal layers. (B) In contrast, in mixed biofilms with *C. albicans*, minimum vancomycin presence or diffusion was seen. (C) Similarly, in biofilms grown with *C. albicans* matrix material, vancomycin presence was limited to the surface of the biofilm, with no or minimal penetration into the biofilm. (D) In biofilms grown in spent medium, there was limited penetration of vancomycin into the biofilm matrix and diffused vancomycin was present in greatly reduced concentrations.

Vancomycin diffusion through single and mixed biofilm matrices was comparatively monitored using fluorescence confocal scanning laser microscopy (CSLM). *S. aureus* biofilms were grown with *C. albicans*, with purified *C. albicans* biofilm matrix material, or in *C. albicans* spent culture medium with secreted material. Formed biofilms were stained with concanavalin A (ConA) to allow visualization of the polysaccharide matrix (red). To visualize vancomycin diffusion through the matrix, a fluorescently tagged vancomycin compound was used. After 1 h, vancomycin (cyan) had significantly penetrated and diffused throughout the single *S. aureus* biofilm matrix, reaching the basal layer (Fig. 8A). In contrast, in the mixed biofilms with *C. albicans*, vancomycin signal was minimally detected, with negligible to no penetration into the matrix seen (Fig. 8B). Similarly, in the biofilms formed with exogenous supplementation with *C. albicans* matrix material, vancomycin was confined to the outer periphery of the biofilm with minimal to no penetration into the biofilm (Fig. 8C), and in biofilms grown in spent medium, only limited vancomycin penetration into the biofilm was seen (Fig. 8D).

### *S. aureus* cell coating by *C. albicans* purified and secreted biofilm matrix components.

**FIG 9:**
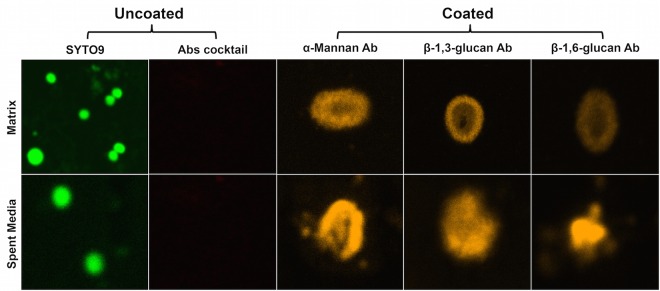
Fluorescence microscopy of *S. aureus* coated with purified and secreted soluble *C. albicans* biofilm matrix material. *S. aureus* planktonic cultures were grown for 24 h in the presence of 0.1 mg/ml of purified matrix or in *C. albicans* spent culture medium. Following washing, cells were treated with α-mannan-, β-1,3-glucan-, and β-1,6-glucan-specific antibodies (Abs) (30 min); stained with a secondary antibody (30 min); and then examined by CSLM. Images revealed the presence of all three matrix components around the bacterial cells when grown with purified matrix material. No fluorescence was detected in control cells from biofilm grown without matrix. Control cells grown in the absence of matrix did not fluoresce but could be visualized by Syto9 staining. Cells were also grown in *C. albicans* spent culture medium (no matrix supplementation) in order to assess whether secreted matrix components also coat bacterial cells. Similar to what was seen with the purified matrix, cells fluoresced when labeled with the antibodies. Control cells grown in fresh medium did not fluoresce but could be seen by Syto9 staining.

In order to evaluate whether *C. albicans* matrix polysaccharides also coat the bacterial cells, *S. aureus* planktonic cell suspension was incubated with *C. albicans* purified matrix and coating was assessed using monoclonal antibodies specific to each of the *C. albicans* matrix components. Using fluorescence microscopy, images demonstrated rapid coating of all 3 matrix components (α-mannan, β-1,3-glucan, and β-1,6-glucan) as assessed by the presence of fluorescence around the bacterial cells. *S. aureus* cells not treated with matrix demonstrated no fluorescence when labeled with antibodies. In addition to purified matrix, to determine whether the secreted matrix material similarly coats the bacterial cells, experiments were also performed where *S. aureus* was incubated in the *C. albicans* spent culture medium. Labeling of bacterial cells with the three specific antibodies demonstrated the presence of all matrix components around the surface of *S. aureus* cells (Fig. 9).

### Enhanced tolerance of *S. aureus* in mixed biofilm is not antibiotic, methicillin resistance, or *S. aureus* strain dependent.

In order to determine whether the *C. albicans*-conferred enhanced tolerance of *S. aureus* in mixed biofilm is exclusive to vancomycin- or methicillin-resistant strains of *S. aureus*, susceptibility assays were also performed using two additional antibiotics, oxacillin and nafcillin. Further, the susceptibility of a methicillin-susceptible *S. aureus* (MSSA) strain was also tested against all 3 antibiotics in biofilms with *C. albicans*. Based on *S. aureus* CFU recovery, results demonstrated that, similarly to vancomycin, the MRSA strain exhibited enhanced tolerance to both oxacillin and nafcillin in mixed biofilm (see Fig. S1 in the supplemental material). A comparable increase in tolerance to all three antibiotics was also seen with the MSSA strain (data not shown).

## DISCUSSION

Traditional therapies for biofilm-associated infections involve removal of infected devices, in addition to multidrug administration that is generally aimed at individual causative agents without consideration for effect on a polymicrobial cause. However, the medical community is recognizing the significance of polymicrobial diseases, and many therapies are now taking into account the cause of these conditions and the repercussions for treatment and prevention (42). Therefore, understanding the physical and molecular interactions between diverse microorganisms will greatly aid in defining new strategies for disrupting these complex mixed infections (20, 30, 33, 43).

Production of extracellular matrix polysaccharides is considered one of the key resistance mechanisms in microbial biofilms, and recent efforts have been focused on understanding the genetic basis for how biofilm matrix production governs drug resistance (16). In exploring the influence of *C. albicans* biofilm matrix on antifungal susceptibility, studies by Nett et al. (44) demonstrated that the matrix sequestered azole antifungal drugs and prevented them from reaching their target**.** In this study, we sought to demonstrate that *C. albicans* matrix may similarly confer protection on other microbial species against their respective antimicrobials when coexisting within mixed biofilms. Specifically, we explored the efficacy of antibacterial drugs against *S. aureus* in mixed fungal-bacterial biofilms, focusing on the role of the *C. albicans* β-1,3-glucan matrix component.

Our experiments using *C. albicans* strains with inhibition or overexpression of *FKS1* and concomitant variations in matrix glucan amount decreased or increased *S. aureus* susceptibility to vancomycin, respectively (Fig. 3A). It is important to note that the strain with decreased *FKS1* expression used in our experiments is a heterozygous and not a null mutant, as *FKS1* is a vital gene, which makes the observed decrease in vancomycin susceptibility with this strain more significant.

Importantly, by supplementing *S. aureus* single-species biofilms with exogenous *C. albicans* biofilm-derived purified matrix material or individual matrix components, we were able to induce the *S. aureus* resistance phenotype in the absence of *C. albicans* (Fig. 2). However, experiments probing the role of matrix mannan using the mannan-degrading enzyme α-mannosidase, and various *C. albicans* mannosylation-deficient mutant strains, yielded no notable difference in *S. aureus* susceptibility to vancomycin. Further evidence for the role of secreted β-1,3-glucan was demonstrated by findings that *C. albicans* spent culture medium enhanced *S. aureus* tolerance to vancomycin; however, the acquired tolerance was significantly diminished when the spent medium was treated with the β-1,3-glucan-degrading enzyme but not the mannan-degrading enzyme (Fig. 3B). These findings are in line of those of Nett et al. (44), where glucanase but not α-mannosidase increased *C. albicans* susceptibility to the antifungal agent fluconazole. Combined, these findings identify β-1,3-glucan as the key matrix component contributing to *S. aureus* vancomycin tolerance in mixed biofilms with *C. albicans*.

Of interest, a study by De Brucker et al. (45) had reported a similar phenomenon involving the tolerance of the Gram-negative bacterial species *Escherichia coli* to the antibiotic ofloxacin in mixed biofilm, and analogously to our findings, β-1,3-glucan was shown to be involved. These observations are interesting in light of our findings that in mixed biofilm, in addition to vancomycin, *S. aureus* also exhibited enhanced tolerance to other antibiotics, namely, oxacillin and nafcillin. Importantly, in testing an MSSA strain with all three antibiotics in mixed biofilm, we also demonstrated that the effect of *C. albicans* on *S. aureus* susceptibility is not *S. aureus* strain dependent or associated with methicillin resistance. Of note and in contrast to our findings, the *C. albicans* zap1Δ/zapΔ mutant strain was shown to provide *E. coli* with increased, albeit minimal, tolerance to ofloxacin.

The *C. albicans* zinc response transcription factor Zap1 was identified by Nobile et al. (46) as a negative regulator of β-1,3-glucan, and the *zap1*Δ*/zap1*Δ strain was found to produce more soluble β-1,3-glucan in biofilms. However, we found no significant difference in *S. aureus* tolerance between growth with this mutant and that with the parental strain (data not shown). These discrepancies are intriguing as Zap1 was also shown to govern synthesis of small, secreted molecules involved in quorum sensing (QS) or interspecies communication. Specifically, Zap1 acts as a positive regulator of the accumulation of farnesol, a QS molecule secreted by *C. albicans* in biofilm, where the *zap1*Δ*/zap1*Δ mutant accumulated significantly less farnesol (46). Interestingly, in a previous study, our findings showed that exogenous synthetic farnesol impacted *S. aureus* susceptibility to various antibiotics, indicating a potential role for this molecule in the *C. albicans*-mediated modulation of the *S. aureus* response to vancomycin (47). Hence, it is conceivable to speculate that any potential notable enhanced effect for the *zap1*Δ*/zap1*Δ mutant on vancomycin response may have been neutralized by the compromised production and secretion of farnesol by the *zap1*Δ*/zap1*Δ mutant. Combined, these observations are of significance as they indicate that QS may also play an important role in mediating the process of enhanced antimicrobial tolerance in biofilm. Therefore, it is important to maintain that although this study focuses on *C. albicans* biofilm matrix components, polymicrobial biofilm formation and antimicrobial resistance are a multifactorial process involving all microbial partners, and thus, the contribution of *S. aureus* to the process cannot be disregarded.

One interesting question is whether the *C. albicans* matrix components, in addition to serving as a barrier, directly bind vancomycin. To explore this possibility, we performed exploratory experiments using the vancomycin antibody in immunoassays to assess polysaccharide binding to vancomycin. However, due to the inherent “stickiness” of the matrix material, these and other experiments were problematic and therefore inconclusive. Nevertheless, the demonstration that the mixed biofilm also conferred protection against other antibacterial agents argues against specific binding to vancomycin being a significant contributor.

Vancomycin is one of the few antibiotics that have remained effective against methicillin-resistant *S. aureus*, and development of resistance to vancomycin is relatively rare (48). Therefore, the demonstration of the failure of vancomycin to effectively penetrate the dense mixed biofilm matrix, as shown in our diffusion imaging (Fig. 8), carries significant clinical implications, as this process may be indicative of a potential therapeutic outcome in a host with a coinfected indwelling medical device. Of similar clinical relevance is the finding that at concentrations subinhibitory to *C. albicans*, the antifungal caspofungin sensitized the matrix-embedded *S. aureus* cells to vancomycin (Fig. 6C).

**FIG 10:**
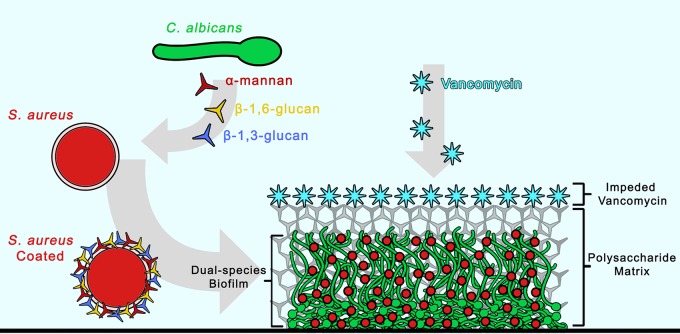
Schematic representation of the proposed “barrier model” as a mechanism for the enhanced *S. aureus* tolerance to vancomycin in mixed biofilm. Based on the combined findings from this study, we propose that the mechanism for the enhanced *S. aureus* tolerance to vancomycin involves impediment of diffusion of antimicrobials through the mixed biofilm matrix consisting of *C. albicans* hyphae and secreted cell wall polysaccharides; α-mannan, β-1,3-glucan, and β-1,6-glucan secreted by *C. albicans* into the mixed biofilm environment adhere to the *S. aureus* cell surface, coating the outer layer. Concurrently, as the biofilm matures, in addition to providing structure and support to the biofilm, the secreted matrix polysaccharides impede the diffusion of antimicrobials into the biofilm interior. Effectively, the polysaccharide matrix acts as a barrier, sequestering vancomycin at the periphery and preventing it from reaching its target.

In conclusion, this study provides compelling evidence demonstrating the therapeutic impact of *C. albicans* cell wall secreted polysaccharide components on a bacterial coinhabitant in a mixed biofilm. To our knowledge, this is the first study utilizing confocal fluorescent time-lapse imaging to visually monitor antimicrobial diffusion through a mixed biofilm. Further, using monoclonal antibodies specific to fungal cell wall components, we demonstrate rapid coating of the bacterial cell by secreted *C. albicans* cell wall polysaccharides. These novel findings are of significance as they may indicate that in addition to hampering diffusion of antimicrobials in a biofilm, the coating of the bacterial cell by fungal secreted polysaccharides may offer the bacteria added protection by preventing the drug from reaching its cellular target (Fig. 10). Therefore, the combined findings from this study warrant awareness in terms of optimizing and overcoming the limitations of current therapies available to treat resilient polymicrobial infections. Importantly, it is crucial to develop suitable animal models to study these phenomena *in vivo*, and such investigations are ongoing in our laboratory.

## MATERIALS AND METHODS

### Reagents.

β-1,3-Glucan (laminarin from *Laminaria digitata*), lyticase from *Arthrobacter luteus* (Zymolyase), mannan (from *Saccharomyces cerevisiae*), concanavalin A (from *Canavalia ensiformis*), α-mannosidase (from jack bean), proteinase K from *Tritirachium album*, and oxacillin sodium and nafcillin sodium were purchased from Sigma-Aldrich Chemical (St. Louis, MO); the MTS tetrazolium-based proliferation assay was from Promega (Madison, WI); vancomycin hydrochloride was from Hospira Inc. (IL, USA); Syto9 green fluorescent nucleic acid stain and vancomycin dipyrromethene boron difluoride (BODIPY) FL conjugate were from Invitrogen (Grand Island, NY); and FUN1 fungal stain was from Thermo Fisher Scientific (Halethorpe, MD).

### Strains and growth conditions.

The standard methicillin-resistant *S. aureus* (MRSA) strain USA300 and the methicillin-susceptible (MSSA) strain ATCC 29213 were used in these studies (49). The following *C. albicans* strains were used where indicated: *C. albicans* reference strains SC5314 (50) and DAY185; heterozygous deletion mutant (*FKS1*/*fks1*Δ) constructed from BWP17, *FKS1*-overexpressing mutant (*TDH3-FKS1*) with one *FKS1* allele under the control of the *TDH3* promoter and one allele intact (18); *zap1*Δ*/zap1*Δ homozygous deletion strain (CJN1201) and the *ZAP1*/*zap1*Δ complemented strain (CJN1193) (46); the *mnn4*Δ (51) and *mnn9*Δ (52) mannosylphosphate transferase mutants with reduced phosphomannan and mannan, respectively; and the *mnt1Δ* and *mnt2Δ* α-1,2-mannosyltransferase mutants and *mnt1Δ*/*mnt2Δ* double mutant (53). *C. albicans* strains were maintained on yeast-peptone-dextrose (YPD) agar (Difco Laboratories) and grown in YPD broth overnight at 30°C with orbital shaking, and cells were equilibrated in fresh medium to an optical density of absorbance of 1.0 at 600 nm. *S. aureus* cultures were grown overnight in Trypticase soy broth (TSB) (Difco) at 37°C and then grown in fresh TSB to mid-log phase. Cells were harvested, washed, and resuspended in RPMI 1640 with l-glutamine and HEPES (Invitrogen, Grand Island, NY) and used at final cell densities of 1 × 10^6^ cells/ml.

### *In vitro* single- and mixed-species biofilm formation.

Biofilms were grown in the wells of 96-well polystyrene flat-bottom plates. *C. albicans* and *S. aureus* cell suspensions were adjusted to 1 × 10^6^ cells/ml in RPMI medium, and 100 µl of cell suspensions was added to the wells individually or in combination. Plates were incubated for 90 min at 37°C, and then wells were washed twice with phosphate-buffered saline (PBS) to remove nonadherent cells. Fresh medium (200 µl) was added to each well, and biofilms were allowed to form for 24 h at 37°C. Following incubation, wells were washed with PBS.

### CV staining of biofilms.

Single and mixed biofilm biomass was quantified using the crystal violet (CV) assay with modifications (54). Biofilms were grown as described above and then washed twice with PBS and air dried at 37°C. Biofilms were stained with 1% aqueous CV solution for 20 min and then washed with sterile water. Plates were air dried, and remaining CV stain was dissolved using 33% acetic acid solution. Following 45 min of destaining, 150 µl of destaining solution was transferred to a new well, and the amount of CV stain was measured with a microtiter plate reader at 595 nm (Titertek; Multiskan MCC1340).

### Biofilm vancomycin susceptibility testing.

The impact of *C. albicans* on the response of *S. aureus* to vancomycin was assessed in biofilms grown as described above. Mixed biofilms were grown using *S. aureus* and each of the *C. albicans* mutant strains for 24 h. Following washing, the wells were supplemented with fresh medium, vancomycin hydrochloride at a final concentration of 800 µg/ml (predetermined based on activity against *S. aureus* single and mixed biofilms) (see Fig. S2 in the supplemental material) was added, and plates were incubated for an additional 24 h at 37°C. Biofilms were then washed twice with PBS, and 100 µl of PBS was added; cells from the biofilms were recovered by sonication followed by vigorous vortexing and pipetting. Cell suspensions were diluted and plated on *C. albicans* and *S. aureus*-specific chromogenic medium (CHROMagar; DRG International, Inc.) for CFU count. Drug-free wells were included as controls. In addition, in order to assess the effect of mixed biofilm growth on *S. aureus* susceptibility to other antibiotics, experiments were also performed using two additional antibiotics, oxacillin (0 to 480 µg/ml) and nafcillin (0 to 160 µg/ml). Further, susceptibility testing using all 3 antibiotics was performed using an alternate strain of *S. aureus* that is susceptible to methicillin (MSSA).

### MTS assay.

Viability was also assessed using the MTS metabolic assay according to the manufacturer’s directions. Following washing with PBS, 100 µl of PBS was added to the wells followed by 20 µl of MTS reagent and plates were incubated at 37°C until color fully developed. Following color development, colorimetric change at 490 nm (*A*_490_) was measured with a microtiter plate reader. On each occasion, reactions were performed in triplicate.

### SEM of single- and mixed-species biofilms.

For SEM analysis, *S. aureus* was grown in single species biofilm with and without exogenous supplementation of purified matrix material (0.5 mg/ml) or glucan (0.25 mg/ml) or with *C. albicans* in mixed biofilms, on coverslips for 48 h. Coverslips were washed twice with PBS and then fixed in 2% paraformaldehyde, 2.5% glutaraldehyde in phosphate buffer, pH 7.4, for 1 h at room temperature and then at 4°C overnight. Following initial fixation, specimens were washed in three changes of 0.1 M PBS for a total of 30 min, postfixed with 1% osmium tetroxide in PBS for 1 h, and washed again in three changes of buffer. Dehydration of specimens was done using a series of graded ethyl alcohol, 30%, 50%, 70%, 90%, and 100% for 10 min each, and two more changes of 100% ethyl alcohol. Specimens were then chemically dried by immersing them sequentially in 2 parts 100% ethyl alcohol-1 part hexamethyldisilazane (HMDS) (Electron Microscopy Sciences, Fort Washington, PA) for 10 min, 1 part 100% ethyl alcohol-1 part HDMS for 10 min, 1 part 100% ethyl alcohol-2 parts HDMS for 10 min, and then 2 changes for 10 min each with 100% HDMS. Specimens were air dried in a hood overnight, mounted on SEM pin mounts, and sputter coated with 10 to 20 nm of platinum-palladium in a sputter coater (EMS 150T ES). SEM images were captured using a Quanta 200 scanning electron microscope (FEI Co., Hillsboro, OR).

### Effect of *C. albicans* purified biofilm-derived matrix material and β-1,3-glucan and mannan exogenous supplementation on *S. aureus* susceptibility to vancomycin in single-species biofilm.

*C. albicans* matrix polysaccharides were extracted from *C. albicans* biofilms and purified as previously described (55). The recovered matrix material (0.25 mg/ml) was used to supplement *S. aureus* biofilms, which were allowed to form for 24 h as described above. *S. aureus* biofilms were also formed in the presence of exogenous β-1,3-glucan (1 mg/ml) or mannan (1 mg/ml) as previously performed (44). Following incubation, biofilms were washed and supplemented with fresh RPMI medium and vancomycin (800 µg/ml), and plates were incubated for an additional 24 h. Following washing, *S. aureus* viability was assessed using the MTS assay and confirmed by CFU recovery for viability.

### Impact of *C. albicans* β-1,3-glucan and mannan enzymatic degradation on *S. aureus* susceptibility to vancomycin in mixed biofilms.

Prior to performing experiments, susceptibility of *C. albicans* to Zymolyase and α-mannosidase was tested to determine the subinhibitory concentrations to be used in mixed biofilm susceptibility testing. Mixed-species biofilms were grown alone or in the presence of glucanase (Zymolyase) (5 U/ml) or α-mannosidase (2 U/ml). Vancomycin (800 µg/ml) was then added alone or in combination with the enzymes, and plates were incubated for an additional 24 h at 37°C. Following incubation, wells were washed three times with PBS and biofilm cells were recovered by sonication and plated for CFU counts. In order to demonstrate lack of contribution of contaminating protease activity in Zymolyase, experiments were also performed using enzymatic digestion with proteinase K.

### Impact of inhibition of *C. albicans* in β-1,3-glucan synthesis on *S. aureus* susceptibility to vancomycin in mixed biofilms.

Mixed 24-h biofilms were treated with the β-1,3-glucan synthase inhibitor caspofungin at final concentrations of 0, 0.06, and 0.5 µg/ml with and without vancomycin (800 µg/ml), and plates were incubated for an additional 24 h. Following washing, biofilms were sonicated and *S. aureus* viability was assessed based on CFU counts. Prior to experiments, susceptibility of *C. albicans* to caspofungin in single species was assessed in order to determine the subinhibitory caspofungin concentration to be used in mixed biofilms. Viability was evaluated using the MTS assay to determine changes in metabolic activity and CFU enumeration for viability.

### Effect of *C. albicans* soluble secreted matrix components on *S. aureus* susceptibility to vancomycin.

In order investigate the role of *C. albicans* secreted matrix components on vancomycin susceptibility, cell-free *C. albicans* biofilm spent culture medium was recovered. Briefly, *C. albicans* (1 × 10^6^ cells/ml) biofilms were grown in 10 ml of RPMI medium in canted-neck flasks and incubated at 37°C for 48 h. Following incubation, spent culture medium was recovered and filter sterilized through a 0.22-µm pore. The filtered medium was then supplemented 1:1 with fresh RPMI medium (prewarmed to 37°C) and used to grow *S. aureus* single-species biofilms. Following 24-h incubation, biofilms were washed and then supplemented with fresh medium and vancomycin (400 µg/ml) (predetermined to be subinhibitory in *S. aureus* single-species biofilm) (see Fig. S2 in the supplemental material) and incubated for an additional 24 h. *S. aureus* viability was assessed using the MTS assay and based on CFU recovery. To identify β-1,3-glucan as the key secreted component in *C. albicans* culture medium, experiments were also performed where *S. aureus* biofilms were cultured in the spent medium (as described above) with the addition of 5 U/ml of the glucan-degrading enzyme glucanase (Zymolyase) for 24 h at 37°C. Following incubation, vancomycin susceptibility testing was performed. Spent medium without glucanase was included as a control. In addition, *C. albicans* biofilms were grown for 24 h and then treated with the β-1,3-glucan synthesis inhibitor caspofungin (0.5 µg/ml) (subinhibitory dose for *C. albicans*) for an additional 24 h. Spent medium was then collected and used in vancomycin susceptibility testing of *S. aureus* single-species biofilms as described above.

### Confocal scanning laser microscopy analysis of vancomycin diffusion through single and mixed biofilms.

To visualize the process of vancomycin diffusion through single and mixed biofilms, confocal scanning laser microscopy was performed as based on a previously described method for *S. aureus* with modifications (48). In addition to monitoring vancomycin diffusion through mixed biofilms, experiments were also performed where *S. aureus* single biofilms were formed in the presence of exogenous supplementation of purified *C. albicans* biofilm matrix to isolate the role of the matrix. Further, as matrix components are secreted during *C. albicans* growth, biofilm spent culture medium was used to grow *S. aureus* biofilms. For these experiments, biofilms were grown on glass coverslip-bottom dishes (MatTek Co., Ashland, MA) in RPMI medium at 37°C for 24 h. Following incubation, biofilms were gently washed three times with PBS and stained with concanavalin A (ConA) for polysaccharide biofilm matrix (100 µg/ml) (red; 488/545). In order to visualize the diffusion of vancomycin, the fluorescent BODIPY FL conjugate of vancomycin (cyan; 488/650) was added to the biofilms to a final concentration of 1 µg/ml. Biofilms were incubated for 1 h and then washed three times with PBS to remove nonpenetrated vancomycin and observed using a 63× oil immersion objective and a Zeiss 710 confocal microscope**.** Images were obtained by LSM 5 Image Browser software at a resolution of 512 by 512 pixels, with an average of 8 images per line. To evaluate the structure and size of the biofilms, a series of images at ≤1-µm intervals in the *z* axis were acquired for the full depth of the biofilm. At least three random fields were visualized for each biofilm, and representative images are presented.

### Fluorescence microscopy analysis of *S. aureus* cell coating with purified and secreted soluble *C. albicans* biofilm matrix material.

In order to examine whether, in addition to their role in biofilms, purified and secreted matrix components also coat the bacterial cell, *S. aureus* cells (2 × 10^7^ cells/ml) were incubated with 0.1 mg/ml of the purified matrix material for 30 min at 37°C. *S. aureus* cells with no matrix treatment were included as controls. In addition to purified matrix, experiments were also performed where *S. aureus* was exposed to *C. albicans* spent culture medium in order to determine whether soluble matrix components secreted by *C. albicans* during biofilm growth similarly coat the bacterial cells. In these experiments, 2 × 10^7^ cells/ml of *S. aureus* were incubated with 1 ml of *C. albicans* spent medium for 30 min at 37°C. *S. aureus* cells incubated in fresh medium were included as a control. Following three washes with PBS, cells were treated with monoclonal antibodies specific to α-mannan (1/100 dilution), β-1,3-glucans (1/6,000 dilution), and β-1,6-glucans (1/4,000 dilution) (produced as previously described) (14) for 1 h at 37°C. Following incubation with primary antibodies, cells were washed three times with PBS and subsequently incubated with a goat anti-mouse IgG Alexa Fluor 488 (orange; 495/519) secondary antibody (1/100 dilution) (Thermo Fisher Scientific) for 30 min at 37°C. Cells were then washed, pelleted, and resuspended in 50 µl PBS. One drop of cell suspension was placed on a slide and covered with a coverslip, and fluorescence was assessed by CSLM. Non-matrix-coated *S. aureus* cells similarly treated with the antibodies were used as a control. However, as control cells with no matrix did not react with antibodies and therefore were not visible, they were stained with Syto9 nucleic acid stain (10 µM) (green; 488/505) in order to be visualized. Cells were observed using a 63× oil immersion objective and a Zeiss 710 confocal microscope**.** Images were obtained by LSM 5 Image Browser software at a resolution of 512 by 512 pixels, with an average of 8 images per line. At least three random fields were visualized for each sample, and representative images are presented.

### Data analysis.

All experiments were performed on at least 3 separate occasions and in triplicate where applicable, and averages were used to present data. All statistical analysis was performed using GraphPad Prism 5.0 software. The Kruskal-Wallis one-way analysis of variance test was used to compare differences between multiple groups, and Dunn’s multiple-comparison test was used to determine whether the difference between two samples was statistically significant. Student’s unpaired *t* test was used to compare differences between two samples. *P* values of ≤0.05 were considered to be significant.

## SUPPLEMENTAL MATERIAL

Figure S1  Assessment of *S. aureus* susceptibility to oxacillin and nafcillin in single *S. aureus* and mixed (CS) biofilms. Preformed (24-h) *S. aureus* single and mixed biofilms were treated with either oxacillin (A) or nafcillin (B) for an additional 24 h. CFU recovery of *S. aureus* from both biofilms showed a significant increase in *S. aureus* recovery from mixed biofilms following oxacillin or nafcillin treatment (**, *P* < 0.01; ***, *P* < 0.001). Means and standard errors of the means are shown. Download Figure S1, TIF file, 0.8 MB

Figure S2  Vancomycin concentration-dependent susceptibility testing in single *S. aureus* (SA) and mixed *S. aureus* and *C. albicans* (SA/CA) biofilms. Preformed (24-h) *S. aureus* single and mixed biofilms were treated with vancomycin (0 to 1,600 µg/ml) for an additional 24 h. Based on the MTS assay, results demonstrated significant and similar *S. aureus* killing activities for all vancomycin concentrations tested in single and mixed biofilms. Vancomycin 800-μg/ml (dual-species biofilms) and 400-μg/ml (single-species biofilms) concentrations were arbitrarily chosen for use in subsequent experiments (**, *P* < 0.01; ***, *P* < 0.001). Download Figure S2, TIF file, 0.5 MB
