# Supplementary figures and images for: Commensal Protection of Staphylococcus aureus against Antimicrobials by Candida albicans Biofilm Matrix
Source: mBio. 2016 Oct 11;7(5):e01365-16. doi: 10.1128/mBio.01365-16 (PMC5061872; doi:10.1128/mBio.01365-16)

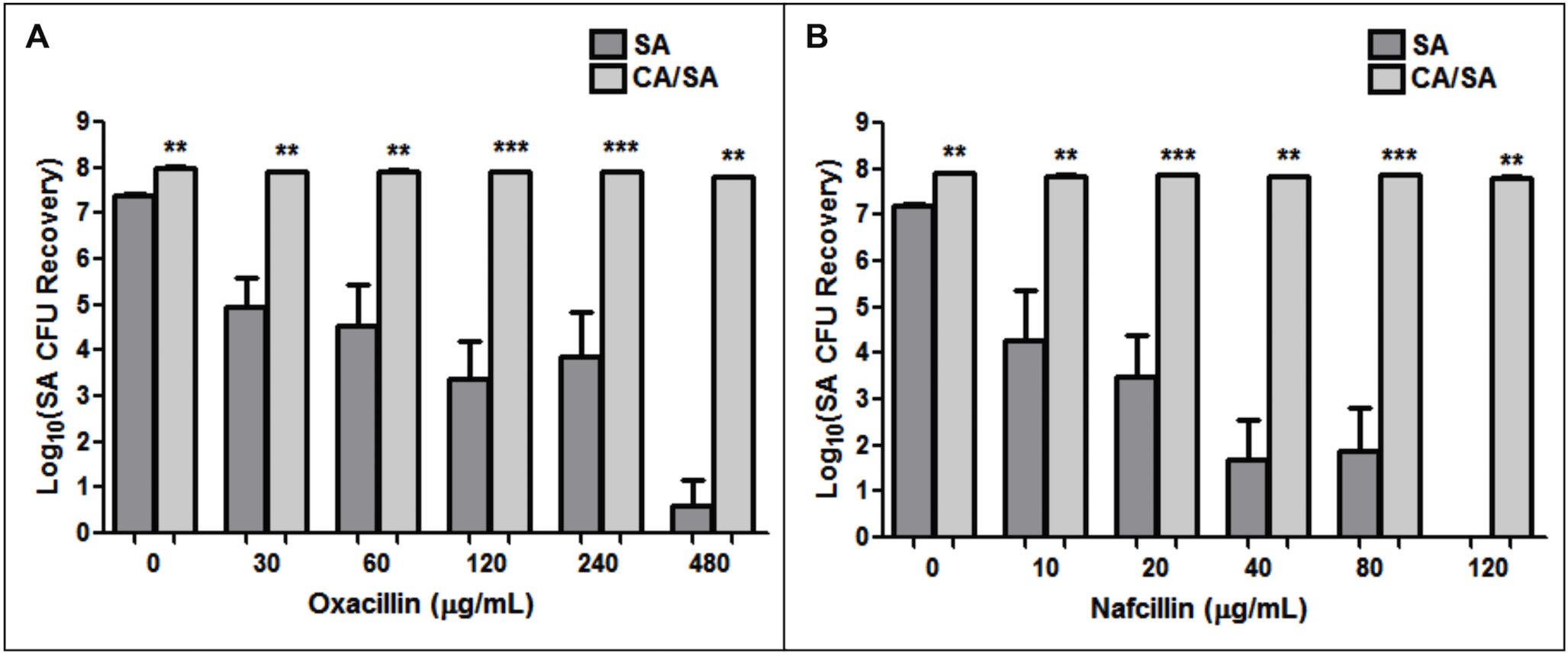

Supplement: Figure S1 — Assessment of S. aureus susceptibility to oxacillin and nafcillin in single S. aureus and mixed (CS) biofilms. Preformed (24-h) S. aureus single and mixed biofilms were treated with either oxacillin (A) or nafcillin (B) for an additional 24 h. CFU recovery of S. aureus from both biofilms showed a significant increase in S. aureus recovery from mixed biofilms following oxacillin or nafcillin treatment (**, P < 0.01; ***, P < 0.001). Means and standard errors of the means are shown. Download [file mbo005163023sf1.tif]

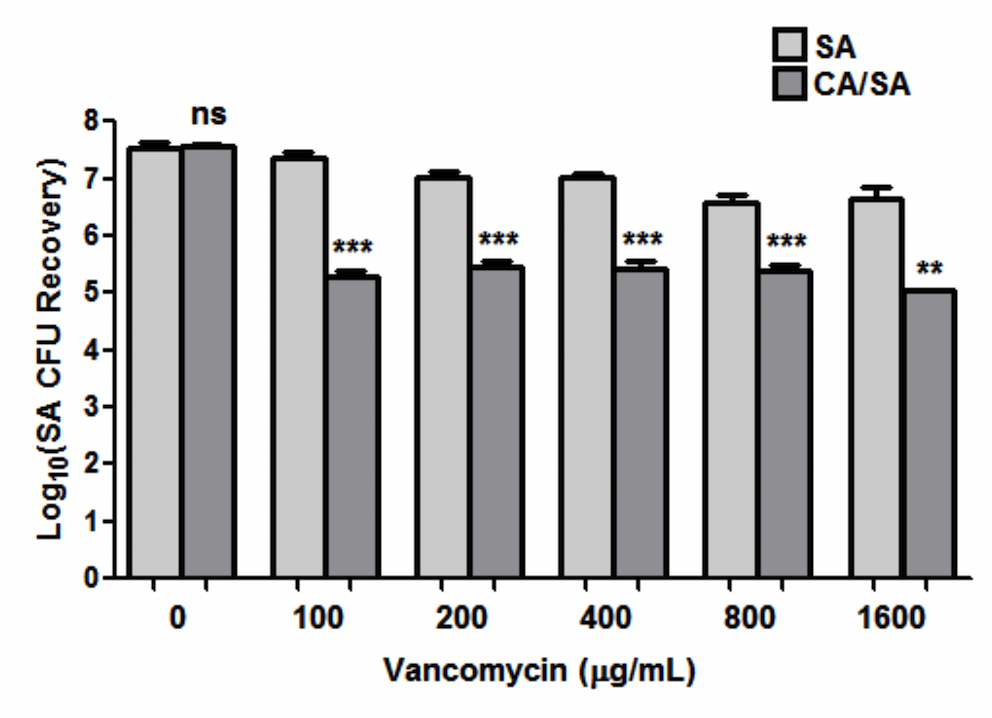

Supplement: Figure S2 — Vancomycin concentration-dependent susceptibility testing in single S. aureus (SA) and mixed S. aureus and C. albicans (SA/CA) biofilms. Preformed (24-h) S. aureus single and mixed biofilms were treated with vancomycin (0 to 1,600 µg/ml) for an additional 24 h. Based on the MTS assay, results demonstrated significant and similar S. aureus killing activities for all vancomycin concentrations tested in single and mixed biofilms. Vancomycin 800-μg/ml (dual-species biofilms) and 400-μg/ml (single-species biofilms) concentrations were arbitrarily chosen for use in subsequent experiments (**, P < 0.01; ***, P < 0.001). Download [file mbo005163023sf2.tif]
